# LncRNA SOX2-OT/miR-30d-5p/PDK1 Regulates PD-L1 Checkpoint Through the mTOR Signaling Pathway to Promote Non-small Cell Lung Cancer Progression and Immune Escape

**DOI:** 10.3389/fgene.2021.674856

**Published:** 2021-07-30

**Authors:** Zhoumiao Chen, Zhao Chen, Shaohua Xu, Qiang Zhang

**Affiliations:** Department of Thoracic Surgery, Sir Run Run Shaw Hospital, School of Medicine, Zhejiang University, Zhejiang, China

**Keywords:** competing endogenous RNA, non-small cell lung cancer, SOX2-OT, mTOR, PD-L1

## Abstract

Non-small cell lung cancer (NSCLC) is the most common type of lung cancer. Currently, treatment methods generally cause poor prognosis. Therefore, in order to seek new treatment options, we explored the internal mechanism of NSCLC. Firstly, the SOX2-OT/miR-30d-5p/PDK1 axis regulated by lncRNA SOX2-OT was predicted by bioinformatics methods, and the expression of SOX2-OT, miR-30d-5p, and PDK1 mRNA in cells were detected by qRT-PCR while PDK1 protein expression was detected by western blot. The results expressed that in NSCLC, SOX2-OT, and PDK1 were notably overexpressed while miR-30d-5p was markedly under-expressed. The interaction between them was verified by dual-luciferase reporter and RNA binding protein immunoprecipitation assays. Subsequently, through CCK8, scratch healing, cell invasion and flow cytometry assays, we revealed that inhibiting the expression of SOX2-OT could inhibit the proliferation, migration and invasion of NSCLC cells and promote cell apoptosis; while simultaneous overexpression of PDK1 or inhibition of miR-30d-5p expression could reverse the inhibitory effect of SOX2-OT silence-mediated malignant progression of NSCLC cells. Then, the combined application of overexpressed PDK1 and rapamycin verified that PDK1 could regulate the expression of PD-L1 in NSCLC cells through the mTOR signaling pathway. Co-culture of CD8^+^ T cells verified that silencing SOX2-OT could inhibit the apoptosis of CD8^+^ T cells through miR-30d-5p/PDK1. Finally, tumor formation assay in animals confirmed that overexpression of SOX2-OT could promote the growth of NSCLC tumor *in vivo*. In this study, assays *in vitro* and *in vivo* were conducted to elucidate the mechanism by which SOX2-OT/miR-30d-5p/PDK1 drives PD-L1 through the mTOR signaling pathway to promote the malignant progression and immune escape of NSCLC.

## Introduction

Lung cancer is the leading cause of cancer-related deaths globally, killing about 1,700,000 people each year. Non-small cell lung cancer (NSCLC) is the most common type of lung cancer, accounting for about 80–90% of all lung cancers (Planchard et al., 2018). For NSCLC patients, after initial diagnosis, corresponding treatment plan should be formulated according to the TNM stage. Surgical treatment is the main treatment for early or partial middle stage NSCLC patients, and chemotherapy alone or radiotherapy combined with chemotherapy is generally given to some middle and advanced NSCLC patients (Miller et al., 2016). Among them, surgical treatment is the best cure, but most of the patients are in the advanced stage of NSCLC at the time of diagnosis and cannot be treated by surgery. In addition, the prognosis of patients undergoing radiotherapy and chemotherapy is generally poor (Hung et al., 2014). Therefore, in recent years, more and more doctors and scholars focus on targeted therapy. In-depth exploration of the internal molecular mechanism of NSCLC can not only provide new therapeutic targets for targeted therapy of NSCLC, but also provide a theoretical basis for the study of prognostic biomarkers of NSCLC.

Among non-coding RNA, long non-coding RNA (lncRNA) is extensively studied as an important RNA that can regulate cancer progression, and can also act as a competing endogenous RNA (ceRNA) to regulate the function of miRNA by competitively binding miRNA response element (MREs) (Chen Z. P. et al., 2018). Abnormal expression of lncRNA may get the regulatory network out of control and eventually lead to the occurrence and progression of cancer (Chan and Tay, 2018). Previous studies showed that lncRNA H19 is highly expressed in NSCLC and regulates NF1 through competitively binding miR-107, thereby promoting the progression of NSCLC (Qian et al., 2018). Down-regulation of lncRNA MALAT1 can suppress the progression of NSCLC by increasing miR-124 and decreasing STAT3 expression (Li et al., 2018). CASC19 sponges miR-130b-3p to modulate ZBR2 as a ceRNA, thus NSCLC progression can be accelerated by regulating the proliferation, migration and invasion of tumor cells (Qu et al., 2019). It can be seen that dysregulation of ceRNA network regulated by lncRNA can have a profound impact on the occurrence and progression of NSCLC, while the regulation of lncRNA SOX2-OT-regulated ceRNA network on the progress of NSCLC has not been reported.

Signaling pathways regulated by lncRNA which functions as a ceRNA are also one of the research directions of the pathogenesis of cancer. Mammalian Target of Rapamycin (mTOR) pathway plays a key regulatory part in physiological processes, such as growth, metabolism, proliferation, metastasis and malignant transformation, of various human tumors (Gomez-Pinillos and Ferrari, 2012). Studies disclosed that when FOXK1 is silenced, the expression level of key proteins in PI3K/AKT/mTOR signaling pathway can be reduced, thus promoting the proliferation and reducing the apoptosis of breast cancer cells (Li Z. Q. et al., 2019). Targeting the activity of PI3K/AKT/mTOR pathway with specific inhibitors can restrain the growth of leukemia cells (Nepstad et al., 2020). Relevant studies on NSCLC found that upregulation of miR-206 can inhibit the activity of PI3K/AKT/mTOR pathway, thus constraining the invasion and migration of NSCLC cells. Therefore, it is extremely crucial to further explore the mTOR pathway when studying the pathogenesis of cancer.

Cancer immune escape is known to be a major obstacle to the design of effective anticancer treatment strategies, and the PD-1/PD-L1 pathway plays a leading role in the tumor immune escape response. Studies uncovered that the expression level of PD-L1 in human ovarian cancer tissue samples is higher than that in adjacent normal tissue. In addition, overexpression of PD-L1 can notably enhance the proliferative ability of ovarian cancer cells (Gao et al., 2019). In hepatocellular carcinoma, anti-PD-L1 therapy can reduce the immune escape of liver tumor cells (Li H. et al., 2019). Cuiling Zhou et al. collected 128 samples of primary NSCLC after surgical resection and tested the expression of PD-L1 by immunohistochemistry, revealing that the overall survival (OS) of the PD-L1 negative expression group was remarkably longer than that of the PD-L1 positive expression group (Zhou et al., 2019). Therefore, both *in vitro* experiments and studies of cancer patient samples showed that PD-L1 plays a vital role in cancer immune escape.

In this study, first of all, the regulatory effect of SOX2-OT as a ceRNA on the malignant progression of NSCLC through miR-30d-5p/PDK1 axis was investigated. Secondly, the driving effect of PDK1 on PD-L1 via the mTOR signaling pathway was explored. In addition, this study also elucidated the mechanism of SOX2-OT/miR-30d-5p/PDK1 axis-mediated immune escape of NSCLC. This paper aimed to explore the internal molecular mechanism of the occurrence and progression of NSCLC, provide a new target for targeted therapy of NSCLC, improve the prognosis of patients and alleviate the suffering of patients.

## Materials and Methods

### Bioinformatics Analysis

Expression data of mature miRNA (Normal: 91, Tumor: 999), lncRNA (Normal: 108, Tumor: 1,037), and mRNA (Normal: 108, Tumor: 1,037) were downloaded from The Cancer Genome Atlas (TCGA) database on March 4th, 2020. Differential analysis was conducted using “edgeR” package on lncRNA, miRNA, and mRNA from normal and tumor group (|logFC| > 1.5, *padj* < 0.05), respectively, to obtain differentially expressed lncRNAs (DElncRNAs), miRNAs (DEmiRNAs), and mRNAs (DEmRNAs). Then, the target lncRNA was identified through literature citation. The expression position of the target lncRNA was determined through lncATLAS (http://lncatlas.crg.eu/). Downstream miRNAs of the target lncRNA were predicted by lncBase (http://carolina.imis.athena-innovation.gr/diana_tools/web/index.php?r=lncbasev2/index-predicted), and the target miRNA was determined by Pearson correlation analysis and literature citation. Next, target mRNA of the target miRNA was predicted by starBase (http://starbase.sysu.edu.cn/index.php) and determined by Pearson correlation analysis and literature citation. Finally, the immune mechanism of the mRNA in NSCLC was determined by mining related literature.

### Cell Culture

All cell lines were derived from BeNa Culture Collection (Beijing, China) and cultured in specific medium. The cell lines used in this study included human normal bronchial epithelial cell line BEAS-2B, human NSCLC cell lines NCI-H460, NCI-H1299, NCI-H292, and A549. The specific cell numbers and corresponding medium are shown in Table 1. All cells were cultured in an incubator at 37°C and containing 5% CO_2_.

**Table 1:** Cell lines used in the study.

**Name**	**Art. No**.	**Medium**
BEAS-2B	BNCC338205	H-DMEM, 90%; FBS, 10%
NCI-H460	BNCC102112	RPMI-1640, 90%; FBS, 10%
NCI-H1299	BNCC351901	RPMI-1640, 90%; FBS, 10%
NCI-H292	BNCC240529	RPMI-1640, 90%; FBS, 10%
A549	BNCC337696	90% F-12K, 90%; FBS, 10%

### Cell Transfection

In cell transfection, miR-30d-5p mimic, miR-30d-5p inhibitor and negative control (NC) were purchased from Sangon Biotech (Shanghai, China). SOX2-OT level was inhibited with short hairpin RNA (shRNA) targeting SOX2-OT, and the control group (sh-NC) was set. The full-length of SOX2-OT and PDK1 were cloned into pcDNA3.1 vector to overexpress SOX2-OT and PDK1, and blank pcDNA3.1 vector was used as control. The vectors were coated with lentivirus and transfected into cells to construct stable cell lines. The synthesized sequences or vectors were transfected into cells with Lipofectamine 2000 (Invitrogen, USA) according to the instructions.

### Real-Time Quantitative Polymerase Chain Reaction (qRT-PCR)

According to the manufacturer's instructions, total RNA was extracted using Trizol Kit (Invitrogen, USA) and complementary DNA (cDNA) was synthesized using PrimeScript 1st Strand cDNA Synthesis Kit (Takara, Kusatsu, Japan). Then the SYBR Green Master Mix II (Takara, Kusatsu, Japan) was used for PCR reaction on ABI PRISM 7500 qRT-PCR Systems (Applied Biosystems, Rockford, IL, USA). GAPDH was the internal reference of lncRNA and mRNA, and U6 was the internal reference of miRNA. Data standardization was based on the 2^−ΔΔCT^ method. Detailed primer sequences are shown in Table 2.

**Table 2:** Primer sequences used in qRT-PCR.

**Gene**		**Primer sequence**
SOX2-OT	Forward primer	5′-CGAAATGGATTCACGGTGCC-3′
	Reverse primer	5′-TGCCAGATCAGGGTGTTGTC-3′
miR-30d-5p	Forward primer	5′-CCTGTTGGTGCACTTCCTAC-3′
	Reverse primer	5′-TGCAGTAGTTCTCCAGCTGC-3′
PDK1	Forward primer	5′-CTCAGGACACCATCCGTTCA-3′
	Reverse primer	5′-ACCATGTTCTTCTAGGCCTTTCAT-3′
PD-L1	Forward primer	5′-TGGCATTTGCTGAACGCATTT−3′
	Reverse primer	5′-TGCAGCCAGGTCTAATTGTTTT-3′
GAPDH	Forward primer	5′-GGACCAATACGACCAAATCCG-3′
	Reverse primer	5′-AGCCACATCGCTCAGACAC-3′
U6	Forward primer	5′-ATGACGTCTGCCTTGGAGAAC-3′
	Reverse primer	5′-TCAGTGTGCTACGGAGTTCAG-3′

### Western Blot

Total proteins were extracted from cultured cells with radio immunoprecipitation assay (RIPA) lysis buffer (Beyotime, Beijing, China). BCA protein assay kit (Pierce, Rockford, IL, USA) was used to determine the protein concentration of cell extracts. Approximately 50 μg protein sample was isolated from each lane by 12% sodium dodecyl sulfate polyacrylamide gel electrophoresis (SDS-PAGE) and then transferred to polyvinylidene fluoride (PVDF) membrane (EMD Millipore, Billerica, MA, USA). The membrane was blocked with 5% skimmed milk powder in TBST buffer solution (20 mM Tris—HCl, pH 7.4, 150 mM NaCl, and 0.1% Tween 20) for 1 h. Then, the membrane was incubated with primary antibodies at 4°C overnight, followed by secondary antibody conjugated with horseradish peroxidase (HRP) at room temperature for 1 h. Protein blot was developed with enhanced electrochemiluminescence (ECL) (GE Healthcare, Piscataway, NJ, NJ). The primary antibodies included rabbit anti-PD-L1 (1:500, ab213524, abcam), rabbit anti-PDK1 (1:1000, ab202468, abcam), rabbit anti-p-mTOR (1:500, ab109268, abcam), rabbit anti-mTOR (1:5,000, ab134903, abcam) and rabbit anti-GAPDH (1:1,500, ab9485, abcam). The secondary antibody was goat anti-rabbit IgG (1:1,000, ab6721, abcam).

### Inhibitor Treatment

After trypsin treatment, transfected NSCLC cells were seeded into 6-well plates. After incubation for 12 h, rapamycin (phosphorylated mTOR (p-mTOR) inhibitor, Selleck Chemicals, Houston, TX, USA;200 nmol/L) were added into 6-well plates and cultured for 24 h. Cells were then collected for qRT-PCR and western blot assays.

### Subcellular Localization Assay

The RNA in the nucleus and cytoplasm of NSCLC cells were isolated using PARIS kit (Thermo Fisher Scientific, USA). qRT-PCR was carried out for quantitative detection of RNA, with U6 as nuclear control and GAPDH as cytoplasmic control.

### RNA Binding Protein Immunoprecipitation (RIP) Assay

The Magna RIP kit (Millipore, Billerica, MA) was implemented for RIP detection. Firstly, NSCLC cells were lysed with RIP lysis buffer and then the lysate of cells was incubated with RIP buffer containing anti-Ago2 (1:15, ab186733, abcam) or IgG (ab172730, 1:100, abcam) antibody coupled magnetic beads. Then the products were digested by protease K buffer followed by RNA purification. Finally, the enrichment of SOX2-OT or miR-30d-5p was determined by qRT-PCR.

### Dual-Luciferase Reporter Assay

The wild-type (WT) or mutated (MUT) 3′-untranslated region (UTR) sequences of SOX2-OT or PDK1 were cloned into the pmir-GLO-promoter vectors (Promega, Madison, USA) to obtain pmirGLO-SOX2-OT-WT (WT-SOX2-OT), pmirGLO-SOX2-OT-MUT (MUT-SOX2-OT), pmirGLO-PDK1-WT (PDK1-WT) and pmirGLO-PDK1- MUT (PDK1-MUT). A549 cells were inoculated into 24-well plates. Then, Lipofectamine 3000 (Invitrogen) kit was employed to transfect WT/MUT-SOX2-OT or PDK1-WT/MUT and miR-30d-5p mimic/mimic NC into A549 cells. The relative luciferase activity was quantified with a dual-luciferase assay kit (Promega) 48 h after transfection.

### Cell Counting Kit-8 (CCK-8)

The cell survival rate was detected by the CCK-8 kit (Yeasen, Shanghai, China) according to the instructions. Transfected A549 cells and NCI-H460 were inoculated into 96-well plates (2,500 cells per well), and 10 μL CCK-8 reagent was added at 0, 24, 48, and 72 h, respectively. After incubation in the dark for 2 h, the optical density (OD) value at 450 nm was determined by a microplate spectrophotometer (ThermoFisher Scientific, USA). Each treatment was set with three repeated wells. The cell activity curve was plotted according to the OD value at 450 nm.

### Scratch Healing Assay

The migration of NSCLC cells was tested by scratch healing assay. Transfected NSCLC cells were inoculated into 6-well plates and cultured for 48 h until the fusion rate reached 90%. An artificial wound was created on the surface of monolayer cells with a 200 μL pipette tip. After washed by PBS, the cells were cultured in serum-free medium. The wound closure was observed by an inverted optical microscope (Axioskop 40, Carl Zeiss AG, Dresden, Germany) at 0 and 24 h. Wound healing rate = (initial width of wound—width of wound 24 h after culture)/initial width of wound ×100%.

### Cell Invasion Assay

The cell invasion assay was carried out on 24-well plates with polycarbonate filter membrane (8 μm pore size, Corning) without polyvinylpyridinoline. The filter membrane was coated with matrix gel (BD Biosciences). Six hundred μL Roswell Park Memorial Institute (RPMI-1640) medium containing 20% fetal bovine serum (FBS) was added to the lower chamber as attractant. NSCLC cell suspensions (5 × 10^4^ cells) from different groups were inoculated into the upper chamber. After incubation at 37°C for 24 h, the uninvaded cells were swabbed gently with a cotton swab while the cells invading to the subface of the membrane were fixed with 4% paraformaldehyde for 30 min and stained with 0.2% gentian violet for 20 min. After washed with PBS 3 times and dried, the cells were observed under a microscope (Axioskop 40, Carl Zeiss AG, Dresden, Germany).

### Flow Cytometry Experiment

The microporous 2-compartment co-culture system was applied for co-culture. Transfected NSCLC cells were inoculated in the upper chamber, while CD8^+^ T (PB009-3-C, ALLCELLS, Shanghai) cells were inoculated in the lower chamber, allowing direct contact between NSCLC cells and immune cells. CD8^+^ T cells were sorted using EasySep™ direct human CD8^+^ T cell isolation kit (STEMCELL, Vancouver, BC, Canada). Annexin V-FITC apoptosis kit (Becton Dickinson, Franklin Lakes, NJ, USA) was used for flow cytometry to determine the percentage of CD8^+^ T cells or apoptotic CD8^+^ T cells according to the manufacturer's instructions.

### Animal Xenotransplantation Assay

The animal research program was approved by the Ethics Committee of animal Research of Sir Run Run Shaw Hospital, School of Medicine, Zhejiang University. All animal experiments were carried out strictly under the supervision and recommendation of the Committee. Six to eight weeks old nude mice were raised in the Animal Research Center of Sir Run Run Shaw Hospital, School of Medicine, Zhejiang University. Each nude mouse was subcutaneously injected with 1 × 106 transfected NSCLC cells. The mice were randomly divided into two groups with six nude mice in each group. Tumor size was recorded once a week for each nude mouse, and tumor volume was calculated as 0.5 × a (length) × b (width). At the end of the study, the nude mice were killed by humanely inhaling carbon dioxide, and the mice that died during the study or did not develop tumors due to other causes or operational problems were excluded. The tumor samples meeting the standard were collected, photographed, and weighed.

### Immunohistochemical Detection

Paraffin-embedded tissue sections were dewaxed in xylene and dehydrated in a series of ethanol solutions. The antigens were extracted using citrate buffer solution (10 mmol/L, pH 6.0) in a microwave oven at 100°C for 15 min. The activity of endogenous peroxidase was blocked by 3% hydrogen peroxide in water for 30 min. The tissue sections were washed with 1 × PBS and pre-sealed with FBS for 30 min. Then, the samples were incubated at 4°C overnight with rabbit anti-PDK1 (1:50, ab227682), rabbit anti-Ki67 (2.5 μg/mL, ab15580) and rabbit anti-PD-L1 (1:150, ab213524), respectively. The sections were sequentially incubated with goat anti-rabbit IgG for 1 h (1:500, ab6721). After washed with 1 × PBS, the tissue sections were incubated with Vectastain ABC reagent (Santa Cruz Biotechnology, Inc., Dallas, TX, USA). DAB substrate solution (Santa Cruz Biotechnology, Inc.) was used for color rendering of immune complexes. Each section was examined under 200× magnification. Finally, the sections were scanned and analyzed using Aperio ImageScope software 12.3 (Leia Biosystems, Wetzlar).

### Data Statistics

The data were obtained from three independent assays, and the experimental results were expressed as mean ± standard deviation. SPSS 19.0 (IBM Corp., Armonk, NY, USA) was used for data analysis. Student's *t*-test was used for comparison between groups while one-way analysis of variance was used to compare the differences between the groups. *P* < 0.05 was considered statistically significant.

## Results

### SOX2-OT Is Highly Expressed in NSCLC Cells and Can Promote the Progression of NSCLC

**Figure 1:**
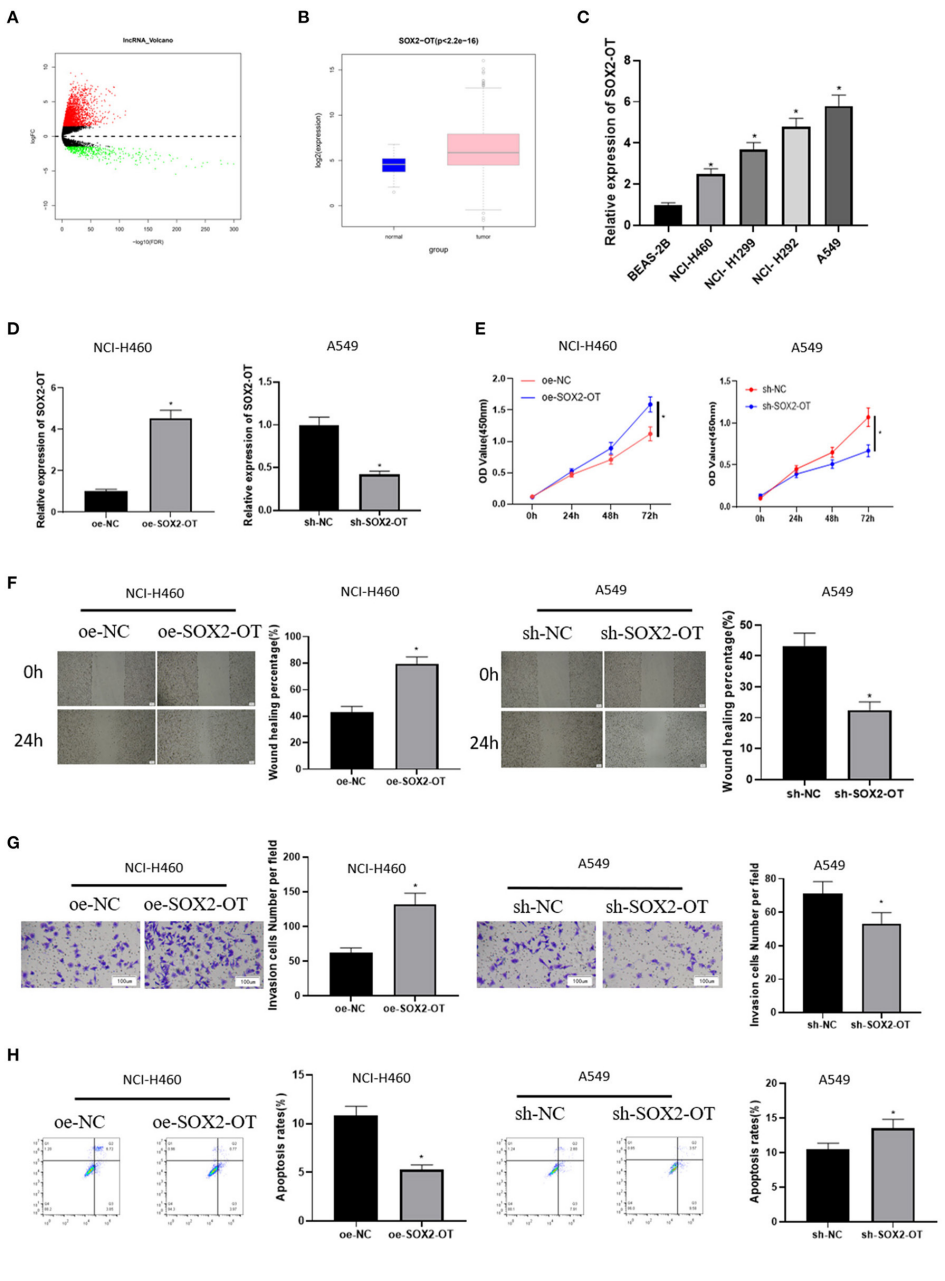
SOX2-OT is highly expressed in NSCLC cells and can promote the progression of NSCLC. **(A)** Volcano map of DElncRNAs between the normal group and the tumor group in the NSCLC data set; **(B)** Expression of SOX2-OT (green box diagram represents normal samples while red box diagram represents tumor samples); **(C)** qRT-PCR detection of SOX2-OT expression in normal cell line BEAS-2B and NSCLC cell lines NCI-H460, NCI-H1299, NCI-H292, and A549; **(D)** Transfection efficiency detected by qRT-PCR; **(E)** Cell proliferation detected by CCK8; **(F)** Cell migration detected by scratch healing assay (40×); **(G)** Cell invasion detected by Transwell assay (100×); **(H)** Cell apoptosis detected by flow cytometry (*p < 0.05).

A total of 2,695 DElncRNAs were obtained by “edgeR” differential analysis (Figure 1A), among which SOX2-OT was proven to be highly expressed in a variety of tumors and to participate in the regulation of ceRNA network (Wo et al., 2019). Therefore, SOX2-OT was selected as the target lncRNA for study. Analysis of SOX2-OT expression levels in different tissues revealed that SOX2-OT was notably highly expressed in NSCLC tumor tissue (Figure 1B). qRT-PCR detection expressed that SOX2-OT expression in NSCLC cell lines was remarkably higher than that of BEAS-2B cell line (Figure 1C), indicating that SOX2-OT had a potential cancer-promoting effect in NSCLC. In order to determine the role of SOX2-OT in the malignant progression of NSCLC, SOX2-OT was overexpressed in NCI-H460 cells which had relatively low expression of SOX2-OT, while SOX2-OT was silenced in A549 cells which had relatively high expression of SOX2-OT. The transfection efficiency was verified by qRT-PCR, showing that the expression level of SOX2-OT was increased markedly in the SOX2-OT overexpressed group, while decreased when silenced (Figure 1D). Cell function experiments results disclosed that in NCI-H460 cells, overexpression of SOX2-OT prominently promoted cell proliferation (Figure 1E), migration (Figure 1F) and invasion (Figure 1G), but inhibited cell apoptosis (Figure 1H). In contrast, SOX2-OT silence produced the opposite effect (Figures 1E–H). These data suggested that SOX2-OT could promote the malignant progression of NSCLC.

### SOX2-OT Targets and Binds to miR-30d-5p in NSCLC Cells

**Figure 2:**
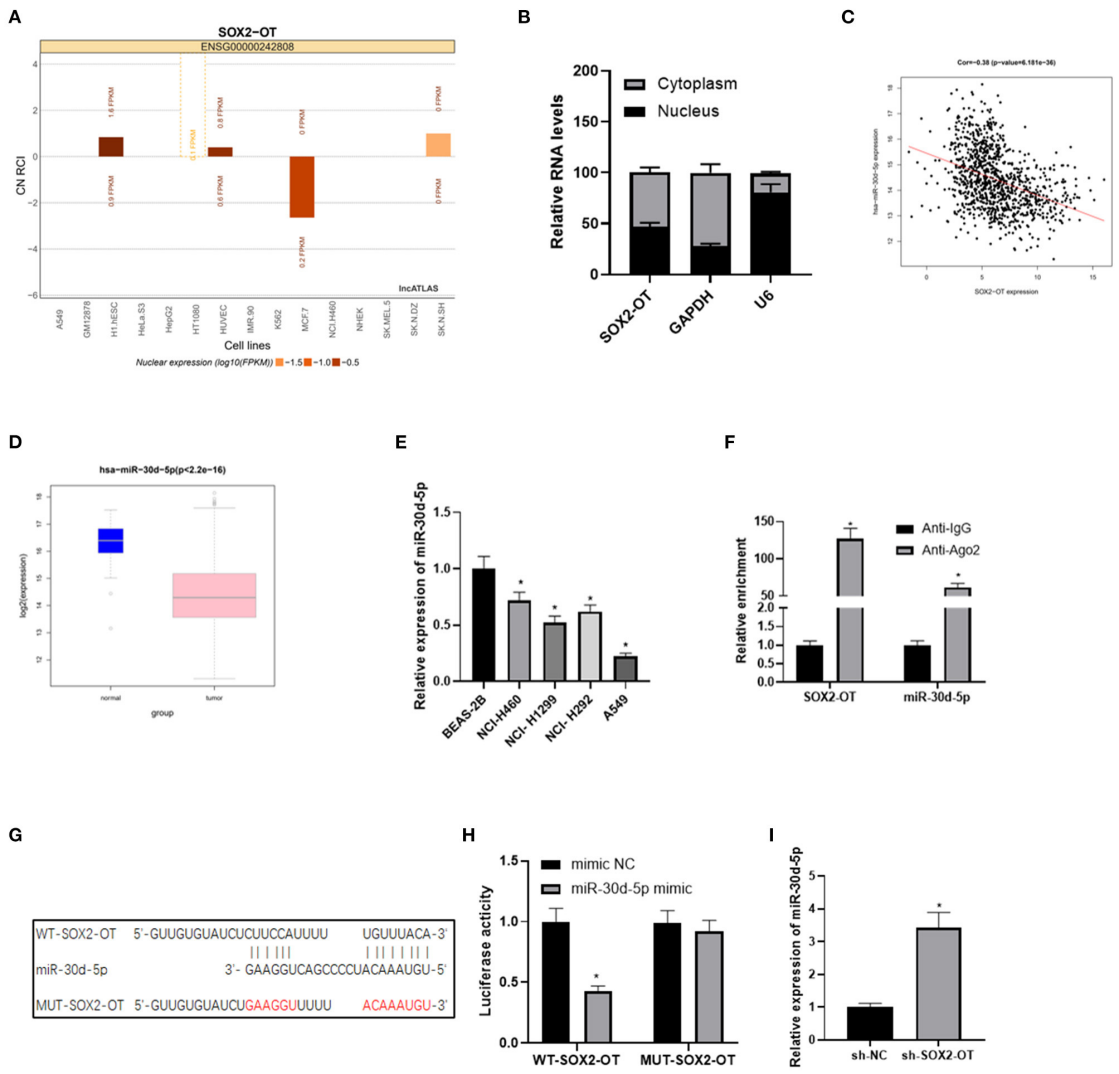
SOX2-OT targets and binds to miR-30d-5p in NSCLC cells. **(A)** LncATLAS website shows SOX2-OT expression in 15 cell lines based on the CN RCI value, with positive value indicating cytoplasm localization and negative value indicating intracellular localization; **(B)** The distribution of SOX2-OT in the cytoplasm and nucleus of NSCLC cells detected by subcellular localization assay; **(C)** Pearson correlation analysis of SOX2-OT and miR-30d-5p; **(D)** Expression of miR-30d-5p (green box plot represents normal samples and red box plot represents tumor samples); **(E)** The expression of miR-30d-5p in normal and NSCLC cells detected by qRT-PCR; **(F)** The interaction between SOX2-OT and miR-30d-5p confirmed by the RIP assay; **(G)** The targeted binding sites between SOX2-OT and miR-30d-5p; **(H)** The targeted binding relationship between SOX2-OT and miR-30d-5p confirmed by dual-luciferase reporter assay; **(I)** The corresponding changes of miR-30d-5p when SOX2-OT was inhibited detected by qRT-PCR (*p < 0.05).

LncATLAS website showed that SOX2-OT was expressed in the cytoplasm and nucleus of 15 cell lines (Figure 2A). We further proved that SOX2-OT was mainly present in the cytoplasm through subcellular localization experiments, indicating that SOX2-OT could participate in the regulation of ceRNA network (Figure 2B). LncBase database was used to predict the downstream target miRNAs of SOX2-OT, and it was found that miR-30d-5p was down-regulated in DEmiRNAs and negatively correlated with SOX2-OT (Figure 2C). According to data analysis based on TCGA, miR-30d-5p was notably lowly expressed in NSCLC tissue (Figure 2D). qRT-PCR results also revealed that miR-30d-5p expression was lower in NSCLC cells compared with that in human bronchial epithelial cells (Figure 2E). Subsequently, RIP assay verified that SOX2-OT and miR-30d-5p could bind in A549 cells (Figure 2F). The targeted binding sequence of SOX2-OT and miR-30d-5p was predicted by the bioinformatics database (Figure 2G). Luciferase reporter assay results showed that after the expression of miR-30d-5p was up-regulated, the luciferase activity was significantly decreased in the WT-SOX2-OT group, while with no obvious change in the MUT-SOX2-OT group (Figure 2H), indicating that SOX2-OT and miR-30d-5p could bind to each other in a targeted way. Finally, after SOX2-OT was inhibited, the expression of miR-30d-5p was found to be increased (Figure 2I). All the above results indicated that SOX2-OT could target and bind to miR-30d-5p in NSCLC cells, and the two were negatively regulated.

### PDK1 Is the Target of miR-30d-5p

**Figure 3:**
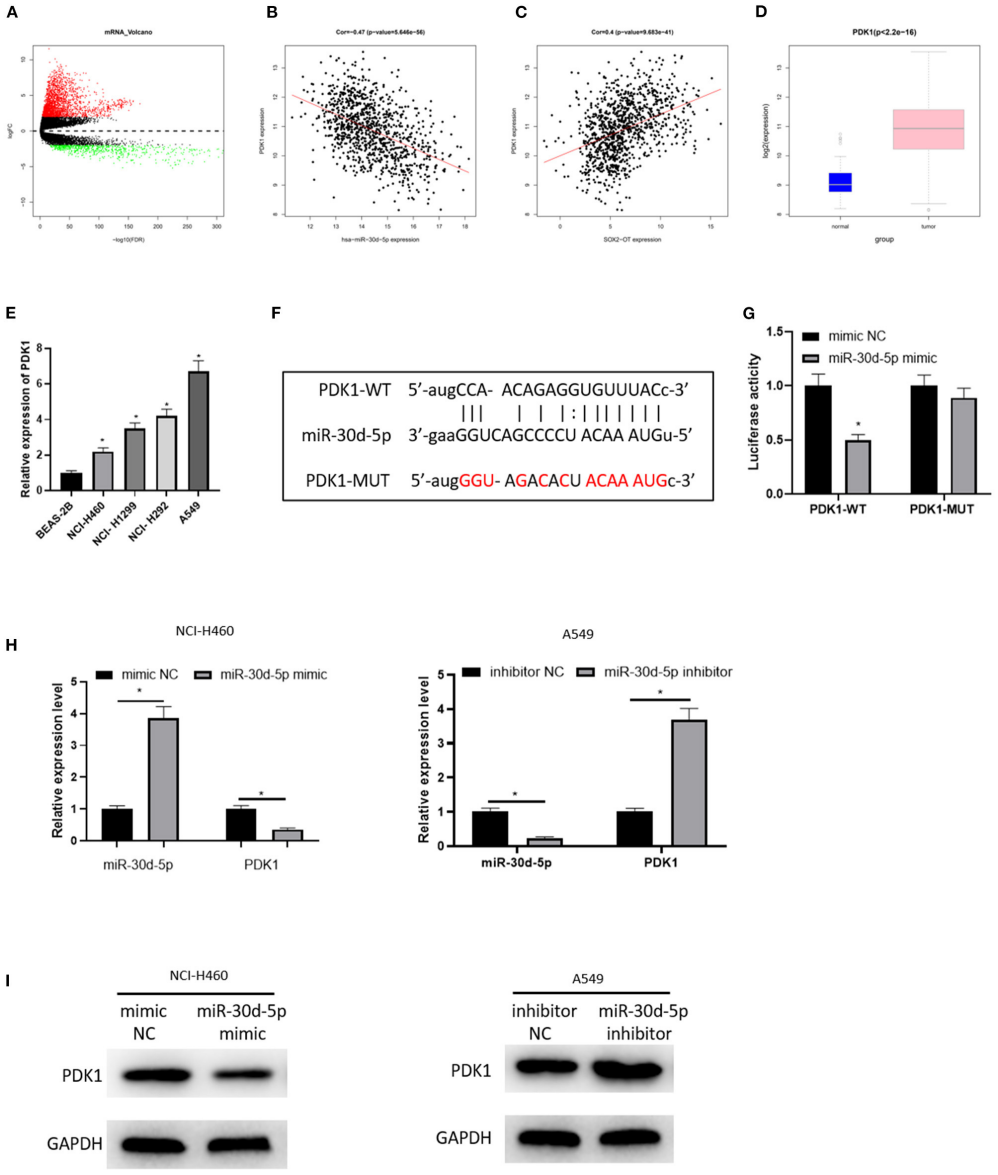
PDK1 is the target of miR-30d-5p. **(A)** Volcano map of DEmRNAs in normal group and tumor group in the NSCLC data set; **(B)** Pearson correlation analysis of PDK1 and miR-30d-5p; **(C)** Pearson correlation analysis of PDK1 and SOX2-OT; **(D)** PDK1 expression in NSCLC tissue (green box diagram represents normal samples and red box diagram represents tumor samples); **(E)** The expression of PDK1 in normal cells and tumor cells detected by qRT-RCR; **(F)** The targeted binding sequence of miR-30d-5p and PDK1 predicted in the bioinformatics database; **(G)** The targeted binding of PDK1 and miR-30d-5p detected by dual-luciferase reporter assay; **(H)** The expression of miR-30d-5p and PDK1 mRNA when miR-30d-5p was abnormally expressed detected by qRT-RCR; **(I)** The protein expression of PDK1 when miR-30d-5p was abnormally expressed detected by western blot (*p < 0.05).

Then, the downstream target of miR-30d-5p was further excavated. 2,950 DEmRNAs were obtained by “edgeR” differential analysis (Figure 3A), revealing that PDK1 was up-regulated in the DEmRNAs and negatively correlated with miR-30d-5p (Figure 3B), and positively correlated with SOX2-OT (Figure 3C). Analysis of the expression levels of PDK1 in different tissues showed that PDK1 was highly expressed in NSCLC cancer tissue (Figure 3D). The expression of PDK1 in human bronchial epithelial cell line BEAS-2B and NSCLC cell lines was detected by qRT-PCR, uncovering that PDK1 was notably highly expressed in NSCLC cells (Figure 3E), which was the opposite of the expression of miR-30d-5p in NSCLC. In addition, the starBase database was implemented to predict the target mRNA of miR-30d-5p, finding that miR-30d-5p and PDK1 had targeted binding sequences (Figure 3F). The dual-luciferase reporter assay also uncovered that with the up-regulated expression of miR-30d-5p, the luciferase activity was remarkably decreased in PDK1-WT group, while did not significantly change in PDK1-MUT group (Figure 3G). qRT-PCR and western blot results indicated that PDK1 expression was decreased when miR-30d-5p was overexpressed but increased when miR-30d-5p was silenced (Figures 3H,I). All the above results suggested that PDK1 was the downstream target of miR-30d-5p, and miR-30d-5p negatively regulated the expression of PDK1.

### SOX2-OT Promotes the Progression of NSCLC by Regulating miR-30d-5p/PDK1 Axis

**Figure 4:**
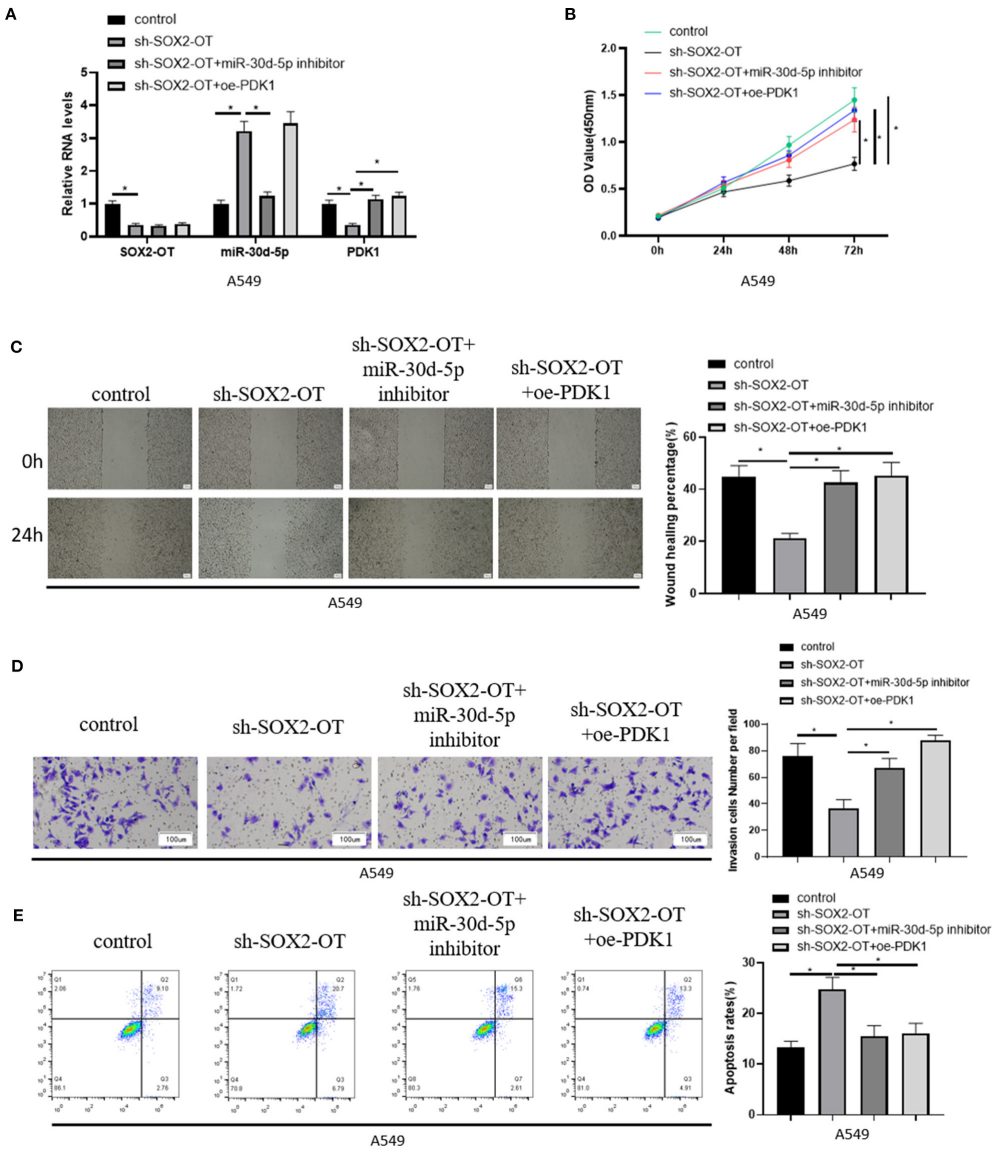
SOX2-OT promotes the progression of NSCLC by regulating miR-30d-5p/PDK1 axis. **(A)** The expression of SOX2-OT, miR-30d-5p and PDK1 mRNA in groups of control, sh-SOX2-OT, sh-SOX2-OT + miR-30d-5p inhibitor and sh-SOX2-OT + oe-PDK1 detected by qRT-RCR; **(B)** The proliferation of A549 cells in each group detected by CCK8; **(C)** The migration of A549 cells in each group detected by scratch healing assay (40×); **(D)** The invasion of A549 cells in each group detected by Transwell assay (100×); **(E)** The apoptosis of A549 cells in each group detected by flow cytometry (*p < 0.05).

To further demonstrate the mechanism of SOX2-OT targeting miR-30d-5p/PDK1 axis and its effect on NSCLC, A549 cells in each group were treated and divided into control group, sh-SOX2-OT group, sh-SOX2-OT + miR-30d-5p inhibitor group and sh-SOX2-OT + oe-PDK1 group, respectively. The expression of SOX2-OT, miR-30d-5p and PDK1 mRNA in each group were detected by qRT-PCR. The results expressed that when SOX2-OT was silenced alone, the expression of miR-30d-5p was increased while the expression of PDK1 was decreased, which again confirmed the negative regulatory relationship between SOX2-OT and miR-30d-5p, and between miR-30d-5p and PDK1 (Figure 4A). Then, cell proliferative, migratory and invasive abilities were detected by CCK8 assay, scratch healing assay and cell invasion assay, respectively. It was found that overexpression of PDK1 or inhibition of miR-30d-5p expression could restore the inhibitory effect of SOX2-OT silence-mediated malignant progression of NSCLC cells (Figures 4B–D). Flow cytometry results indicated that, compared with silencing SOX2-OT alone, overexpressing PDK1 or inhibiting miR-30d-5p while silencing SOX2-OT could reduce the apoptotic rate of NSCLC cells (Figure 4E). The above results proved that SOX2-OT could promote the progression of NSCLC by regulating the miR-30d-5p/PDK1 axis.

### PDK1 Can Regulate the Expression of PD-L1 in NSCLC Cells Through mTOR Signaling Pathway

**Figure 5:**
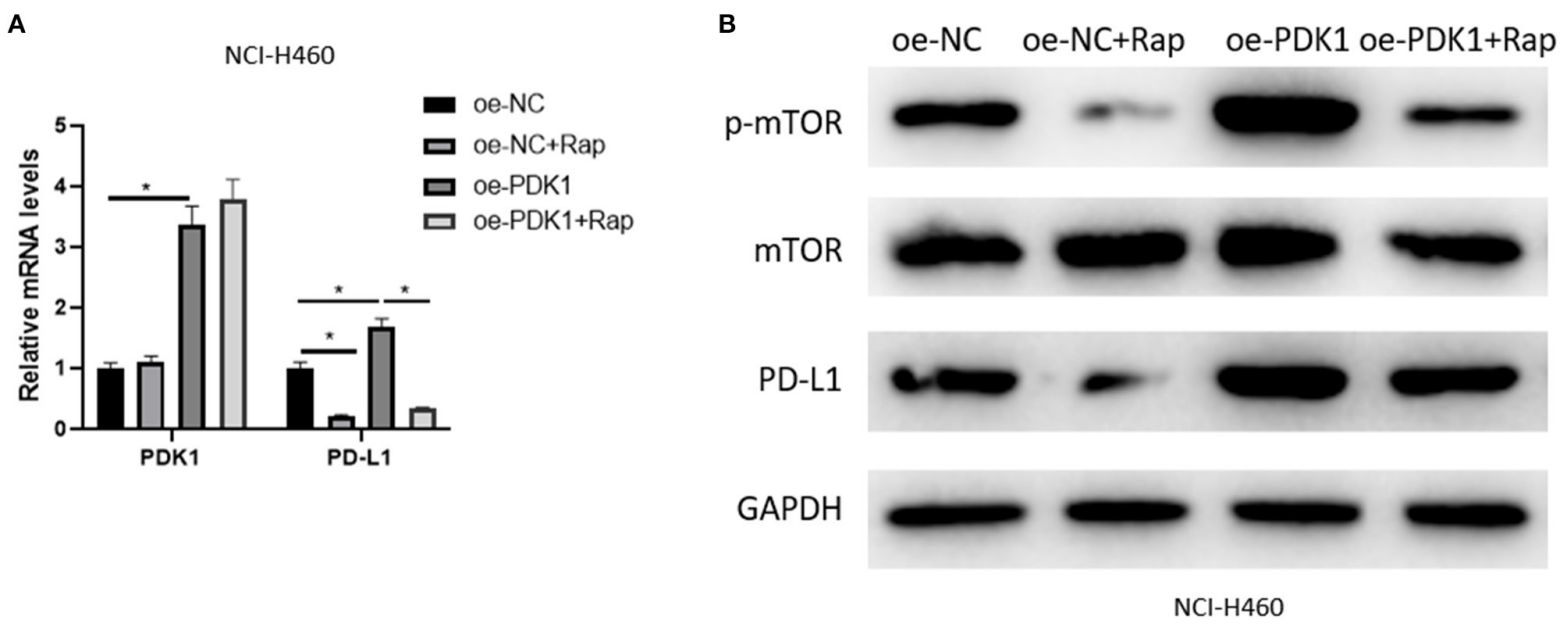
PDK1 can regulate the expression of PD-L1 in NSCLC cells through the mTOR signaling pathway. **(A)** The mRNA expression of PDK1 and PD-L1 detected by qRT-PCR; **(B)** The protein expression of p-mTOR, mTOR, and PD-L1 detected by western blot (*p < 0.05).

The mechanism of SOX2-OT/miR-30d-5p/PDK1 axis on NSCLC cells was studied above, and it was confirmed that PDK1 acts on mTOR signaling pathway in NSCLC (Chen G. M. et al., 2018; Liu et al., 2019) and the activation of the pathway can drive PD-L1 in NSCLC (Lastwika et al., 2016). Therefore, in order to explore the regulation of PDK1 through mTOR signaling pathway on PD-L1 in NSCLC cells, we overexpressed PDK1 in NCI-H460 cell line, and the mTOR signaling pathway was inhibited by Rapamycin. Then the expression of PD-L1 was detected in the oe-NC group, oe-NC + Rapamycin (Rap) group, oe-PDK1 group and oe-PDK1 + Rap group. The results suggested that Rapamycin reduced the expression of phosphorylated mTOR (p-mTOR) and PD-L1 compared with the control group, and the overexpression of PDK1 promoted the expression of p-mTOR and PD-L1. Compared with the oe-PDK1 group, the combined use of Rapamycin reduced the previously up-regulated expression of p-mTOR and PD-L1 (Figures 5A,B). In conclusion, PDK1 could regulate the expression of PD-L1 in NSCLC cells through the mTOR signaling pathway.

### SOX2-OT Can Promote Apoptosis of CD8^+^ T Cells Through miR-30d-5p/PDK1

**Figure 6:**
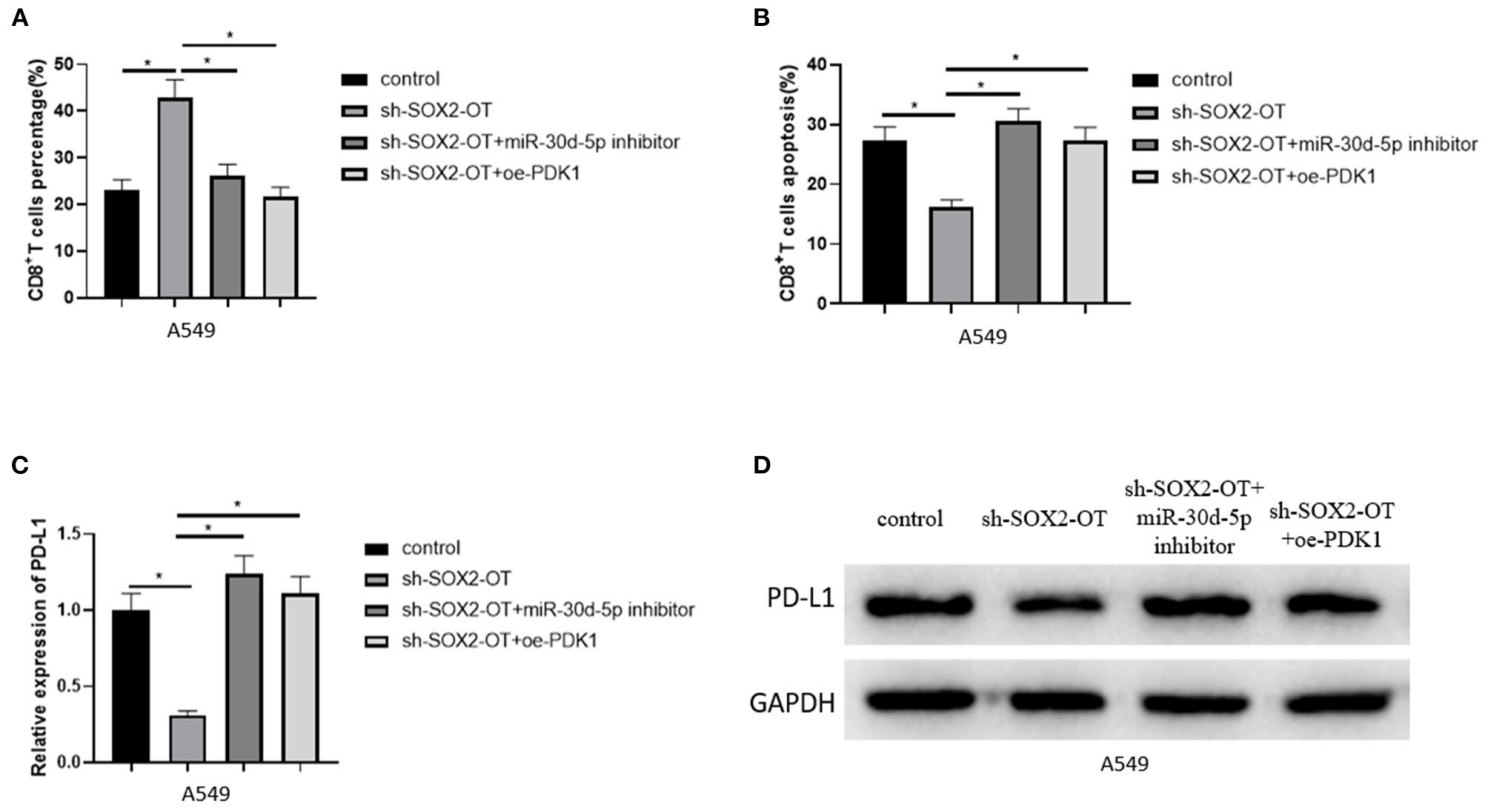
SOX2-OT can promote apoptosis of CD8^+^T cells through miR-30d-5p/PDK1. **(A)** The percentage of CD8^+^ T cells in each group detected by flow cytometry; **(B)** The percentage of apoptotic CD8^+^ T cells in each group detected by flow cytometry; **(C)** The expression of PD-L1 in each group detected by qRT-PCR; **(D)** The expression of PD-L1 in each group detected by western blot (*p < 0.05).

To clarify the mechanism of NSCLC immune escape, we simulated the tumor microenvironment and co-cultured transfected A549 cells with CD8^+^ T cells and analyzed the percentage of CD8^+^ T cells and the apoptotic rate of CD8^+^ T cells by flow cytometry. The results revealed that when SOX2-OT was silenced in A549 cells, the percentage of CD8^+^ T cells was increased and the percentage of CD8^+^T cell apoptosis was decreased. When SOX2-OT and miR-30d-5p were silenced simultaneously, or when SOX2-OT was silenced and PDK1 was overexpressed, the percentage of CD8^+^ T cells and the percentage of CD8^+^ T cell apoptosis were restored (Figures 6A,B). In addition, we also detected the expression of PD-L1 in each group of cancer cells by qRT-PCR and western blot. The results uncovered that the expression of PD-L1 was decreased when SOX2-OT was silenced alone compared with the control group. Compared with the sh-SOX2-OT group, when SOX2-OT and miR-30d-5p were silenced at the same time, or when SOX2-OT was silenced while PDK1 was overexpressed, the expression of PD-L1 was reversely increased (Figures 6C,D). Thus, SOX2-OT could promote the expression of PD-L1 through miR-30d-5p/PDK1 axis and thus cause the immune escape of NSCLC.

### SOX2-OT Can Promote the Growth of NSCLC Tumor *in vivo*

**Figure 7:**
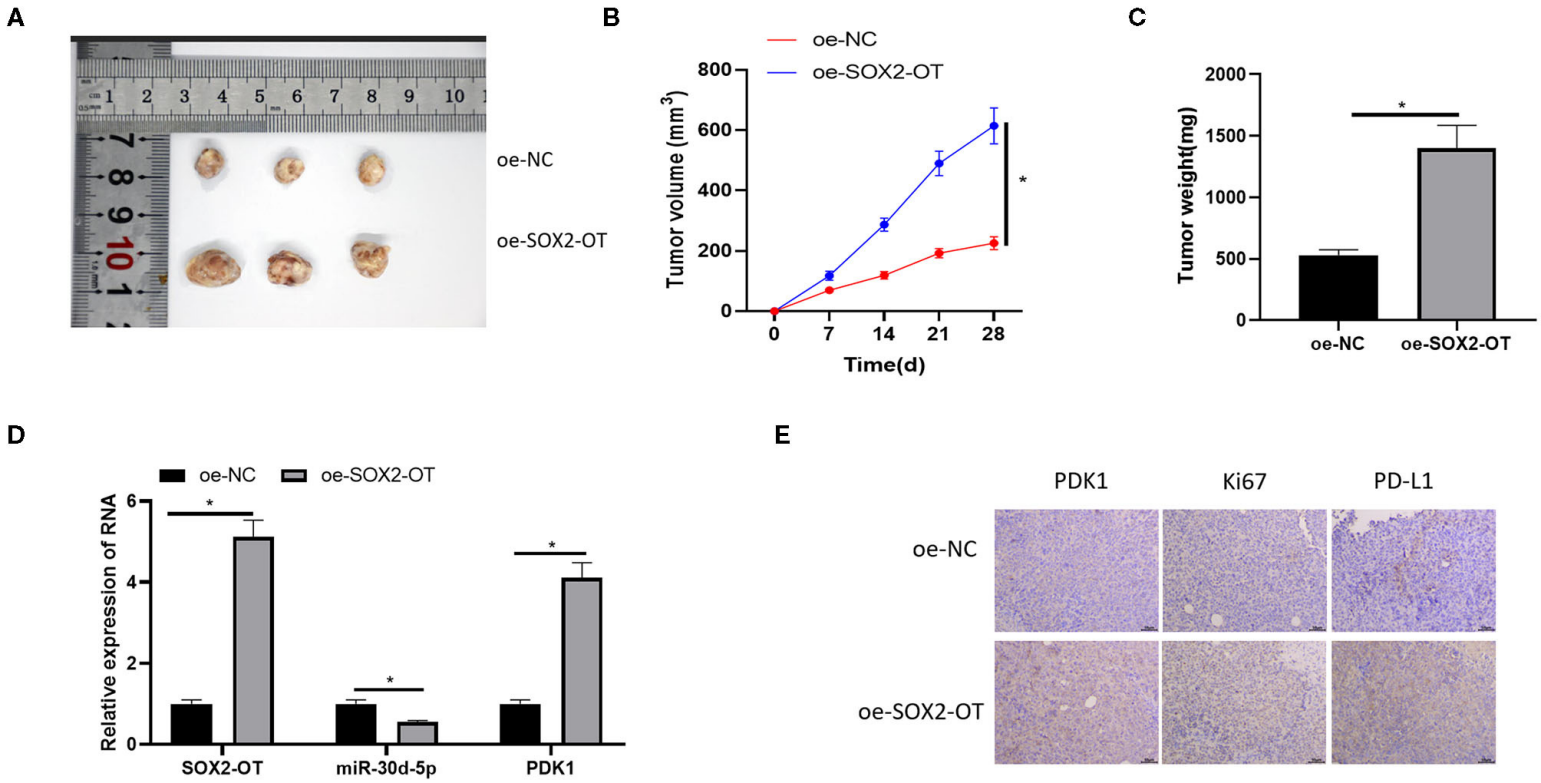
SOX2-OT can promote the growth of NSCLC tumor *in vivo*. **(A)** Representative tumor images; **(B)** Line diagram of tumor volume change; **(C)** Histogram of tumor weight; **(D)** The expression of SOX2-OT, miR-30d-5p, and PDK1 upon SOX2-OT overexpression detected by qRT-PCR; **(E)** The expression of PDK1, Ki67 and PD-L1 in each group detected by immunohistochemistry (*p < 0.05).

Finally, the effect of SOX2-OT on tumorigenicity of NSCLC was tested in nude mice. It was observed that overexpression of SOX2-OT resulted in increased tumor volume and weight in mice (Figures 7A–C). qRT-PCR detected the expression of SOX2-OT, miR-30d-5p and PDK1 in tumor cells of each group, indicating that overexpression of SOX2-OT could down-regulate miR-30d-5p and up-regulate PDK1 expression (Figure 7D). Subsequently, tumor tissue sections were prepared in each group, and the protein expression of PDK1, Ki67 and PD-L1 were detected by immunohistochemistry. Results revealed that overexpression of SOX2-OT increased the expression of PDK1, Ki67 and PD-L1 compared with the control group (Figure 7E). Taken together, SOX2-OT could promote the tumor growth of NSCLC.

## Discussion

More and more studies show that lncRNA is involved in the regulation of the malignant process of cancer. For example, lncRNA PTCSC3 can negatively regulate the proliferation, invasion and migration of gastric cancer cells (Zhang et al., 2020). High expression of lncRNA SNHG11 promotes proliferation and metastasis of colorectal cancer cells by targeting the Hippo pathway (Xu et al., 2020). In this paper, we found that lncRNA SOX2-OT was markedly up-regulated in NSCLC through bioinformatics analysis and detection of corresponding expression at the cellular level, which meant that SOX2-OT was involved in the regulation of the malignant process of NSCLC. However, no relevant results of SOX2-OT in NSCLC were found in previous studies. This study was the first to report the expression of SOX2-OT and its specific regulatory mechanism in NSCLC, and it was found that overexpression of SOX2-OT would promote the proliferation, migration and invasion and reduce the apoptosis of NSCLC cells.

LncRNA is known to act as miRNA sponge. For example, SOX2-OT accelerates cell proliferation and migration in prostate cancer by targeting the miR-369-3p/CFL2 axis (Wo et al., 2019). SOX2-OT knockdown inhibits the growth of prostate cancer *in vivo* by regulating the miR-452-5p/HMGB3 axis (Song et al., 2020). In this study, the target downstream miRNA of SOX2-OT and that of the miRNA were predicted by bioinformatics methods, and a ceRNA network, SOX2-OT/miR-30d-5p/PDK1, regulated by SOX2-OT was obtained. A study showed that overexpression of miR-30d-5p inhibits the proliferation, migration and invasion of NSCLC cells (Gu et al., 2020). PDK1 expression was proven to be up-regulated in NSCLC tissue and cell lines and promoting the expression of miR-330-5p can down-regulate PDK1 and inhibit the growth, migration and invasion of NSCLC cells (Bai et al., 2020). In this study, it was confirmed that SOX2-OT and PDK1 were highly while miR-30d-5p was lowly expressed in NSCLC cells. Moreover, the targeted binding relationships between SOX2-OT and miR-30d-5p, and between miR-30d-5p and PDK1 were also proved by dual-luciferase reporter assay and other assays. In addition, we also conducted rescue experiments to clarify the promoted effect of SOX2-OT/miR-30d-5p/PDK1 axis on the malignant progression of NSCLC.

In addition, a study uncovered that the mTOR pathway, HIF pathway, glycolysis, PI3K/AKT/mTOR signaling pathway, etc. are prominently activated in the PDK1 high expression phenotype of muscular infiltrating bladder urothelial carcinoma (Zhu et al., 2020). The activation of the mTOR signaling pathway can promote the immune escape of cancer by promoting the expression of PD-L1 (Lastwika et al., 2016). Therefore, through the combined use of Rapamycin, we proved that PDK1 could regulate the expression of PD-L1 in NSCLC cells through the mTOR signaling pathway. In addition, it is established that tumor cells can change the activity of T cells to avoid the anti-tumor immune response, thus promoting the survival of tumor cells (Vinay et al., 2015; Lu et al., 2017). Tumor cells can also interact with CD8^+^ T cells and induce apoptosis, thus promoting tumor progression (He et al., 2017; Konen et al., 2019). Therefore, subsequently, we interpreted the immune escape of NSCLC cells mediated by PD-L1 and found that the expression of PD-L1 was decreased when SOX2-OT was silenced, and the co-culture results with CD8^+^ T cells revealed that silencing SOX2-OT would increase the percentage of CD8^+^ T cells and reduce the percentage of apoptotic cells. In other words, SOX2-OT could promote the immune escape of NSCLC by regulating the expression of PD-L1.

In this study, we demonstrated that SOX2-OT was notably highly expressed in NSCLC and could promote the malignant progression of NSCLC, and also clarified the targeted regulatory relationship between SOX2-OT, miR-30d-5p, and PDK1. In addition, this study also explained the mechanism of SOX2-OT-mediated immune escape in NSCLC and verified the driving effect of SOX2-OT/miR-30d-5p/PDK1 axis on PD-L1 through the mTOR signaling pathway. Finally, we verified that SOX2-OT could promote the growth of NSCLC *in vivo* through tumor formation assay in nude mice. These findings provide new insights into the underlying molecular mechanisms of the occurrence and progression of NSCLC and also prove that SOX2-OT is a new target for the treatment of NSCLC with broad application prospects. However, this study also has some limitations, such as the failure to verify the expression levels of SOX2-OT, miR-30d-5p, and PDK1 in the tissue of NSCLC patients at the clinical level. In the future, we will enrich its expression in clinical samples.

## Data Availability Statement

The datasets presented in this study can be found in online repositories. The names of the repository/repositories and accession number(s) can be found in the article/supplementary material.

## Ethics Statement

This animal study was reviewed and approved by Ethics Committee of animal Research of Sir Run Run Shaw Hospital, School of Medicine, Zhejiang University.

## Author Contributions

ZhaC contributed to the study design. SX conducted the literature search and acquired the data. ZhoC wrote the article and gave the final approval of the version to be submitted. QZ performed data analysis and drafted and revised the article. All authors contributed to the article and approved the submitted version.

## Conflict of Interest

The authors declare that the research was conducted in the absence of any commercial or financial relationships that could be construed as a potential conflict of interest.

## Publisher's Note

All claims expressed in this article are solely those of the authors and do not necessarily represent those of their affiliated organizations, or those of the publisher, the editors and the reviewers. Any product that may be evaluated in this article, or claim that may be made by its manufacturer, is not guaranteed or endorsed by the publisher.
