# Corrigendum: LncRNA SOX2-OT/miR-30d-5p/PDK1 Regulates PD-L1 Checkpoint Through the mTOR Signaling Pathway to Promote Non-Small Cell Lung Cancer Progression and Immune Escape

**DOI:** 10.3389/fgene.2022.857986

**Published:** 2022-03-03

**Authors:** Zhoumiao Chen, Zhao Chen, Shaohua Xu, Qiang Zhang

**Affiliations:** Department of Thoracic Surgery, Sir Run Run Shaw Hospital, School of Medicine, Zhejiang University, Hangzhou, China

**Keywords:** non-small cell lung cancer, competing endogenous RNA, SOX2-OT, mTOR, PD-L1

There is an error in the Funding statement. The correct number for Zhejiang Provincial Natural

Science Foundation of China “No. Q19H280028” is “No. LQ19H280007.”

The authors apologize for this error and state that this does not change the scientific conclusions of the article in any way. The original article has been updated.

